# Identifying Higher-Volume Antibiotic Outpatient Prescribers Using Medicare Part D Prescription Data — Arizona, 2021

**DOI:** 10.1017/ash.2024.177

**Published:** 2024-09-16

**Authors:** Swathi Vajrala, Rachana Bhattarai, Tho Pham, Mandana Naderi, Celine Sanchez, Elizabeth Kim, Kenneth Komatsu

**Affiliations:** Arizona Department of Health Services; University of Arizona

## Abstract

**Background:** Increased antibiotic resistance is a rising global health problem that can result from overprescribing and misusing antibiotics. The impact of antibiotic overprescribing is significant, particularly among the older adult population due to increased adverse reactions. The objective of this study is to identify higher volume antibiotic outpatient prescribers and their antibiotic prescription rates by region and provider specialties in Arizona. **Methods:** Publicly available data from the Center for Medicare and Medicaid Services Part D Prescriber database during 2021 was analyzed. Those prescribers who had beneficiaries and antibiotic claims of fewer than 11 were excluded from the analysis. Higher-volume prescribers were identified by estimating the providers who prescribed the highest 10th percentile of antibiotic volume. The cumulative percentage of antibiotic prescriptions and prescriber’s antibiotic volume per 1000 beneficiaries (prescribing rate) were compared between the higher volume prescribers and lower volume prescribers by the EMS region and specialty. Median prescribing rates among prescribers were compared using the Wilcoxon rank-sum test. All analyses were performed using SAS (version 9.4; SAS Institute, NC). **Results:** The number of Arizona prescribers included in the dataset during 2021 was 27,124. After excluding prescribers with fewer than 11 antibiotic prescriptions, 14,410 prescribers were included in the analysis. These providers prescribed a total of 1,095,559 antibiotic prescriptions, with a median of 45 antibiotic prescriptions per prescriber. Thirty-nine percent of antibiotic prescriptions were written by the higher volume prescribers and prescribed a median of 236 antibiotic prescriptions. Higher-volume prescribers had a 52% higher median antibiotic prescribing rate compared with lower-volume prescribers (600 versus 396 prescriptions per 1,000 beneficiaries) (p < 0.01). The median antibiotic prescribing rate among higher volume prescribers was highest in the central region (602 antibiotic prescriptions per 1,000 beneficiaries) compared with other regions (581 prescriptions per 1,000 beneficiaries in the north region) (p < 0.01). The top two specialties that higher volume prescribers practiced were family practice and nurse practitioners. Antibiotic prescribing rates of higher-volume prescribers were highest among dentists (1,118 prescriptions per 1,000 beneficiaries).

